# Crosstalk between basal extracellular matrix adhesion and building of apical architecture during morphogenesis

**DOI:** 10.1242/bio.058760

**Published:** 2021-11-29

**Authors:** Mariana Barrera-Velázquez, Luis Daniel Ríos-Barrera

**Affiliations:** 1 Departamento de Biología Celular y Fisiología, Instituto de Investigaciones Biomédicas, Universidad Nacional Autónoma de México, Ciudad Universitaria, Mexico City 04510, Mexico; 2 Undergraduate Program on Genomic Sciences, Centro de Ciencias Genómicas, Universidad Nacional Autónoma de México, Cuernavaca, Morelos 62210, Mexico

**Keywords:** Apicobasal interactions, Cytoskeleton, Dorsal closure, Extracellular matrix, Tracheal development

## Abstract

Tissues build complex structures like lumens and microvilli to carry out their functions. Most of the mechanisms used to build these structures rely on cells remodelling their apical plasma membranes, which ultimately constitute the specialised compartments. In addition to apical remodelling, these shape changes also depend on the proper attachment of the basal plasma membrane to the extracellular matrix (ECM). The ECM provides cues to establish apicobasal polarity, and it also transduces forces that allow apical remodelling. However, physical crosstalk mechanisms between basal ECM attachment and the apical plasma membrane remain understudied, and the ones described so far are very diverse, which highlights the importance of identifying the general principles. Here, we review apicobasal crosstalk of two well-established models of membrane remodelling taking place during *Drosophila melanogaster* embryogenesis: amnioserosa cell shape oscillations during dorsal closure and subcellular tube formation in tracheal cells. We discuss how anchoring to the basal ECM affects apical architecture and the mechanisms that mediate these interactions. We analyse this knowledge under the scope of other morphogenetic processes and discuss what aspects of apicobasal crosstalk may represent widespread phenomena and which ones are used to build subsets of specialised compartments.

## Abstract (precis)

**Summary:** This work analyses the mechanisms that cells use to coordinate cell shape changes at the apical and basal compartments during morphogenesis.

## Introduction

A wide range of mechanisms allow cells to transition from simple cuboidal structures into complex shapes that serve various functions. These mechanisms extend from subcellular, autonomous processes to tissue-scale rearrangements, and they involve complex architectures that stabilise shape changes. Executors of shape changes are, for instance, polarised actomyosin networks that induce apical constriction during *Drosophila melanogaster* gastrulation, or that drive the formation of a leading edge to allow cell migration (Figueiredo et al., 2021; Kolsch et al., 2007; Martin et al., 2009). These changes must be stabilised, and this is achieved through interactions with the extracellular matrix (ECM).

The ECM is a physical and signalling scaffold that allows cells to interact with each other, both in unicellular and multicellular organisms (Chaudhuri et al., 2020; Faria-Oliveira et al., 2015; Hamann and Denness, 2011). The best characterised ECM is the metazoan basal ECM (bECM), whose components constitute ∼2% of the coding genome in vertebrates and in *Drosophila* (Davis et al., 2019). Core bECM constituents are large, fibrillar proteins of the families of Collagen, the proteoglycan Perlecan, and the glycoproteins Laminin and Nidogen. Some of these, like Laminin and Collagen serve as ligands for integrins, which form dimers of α and β subunits and signal through intracellular effectors (Keeley et al., 2020). Therefore, varying bECM structure or composition can greatly influence cell behaviour by modulating integrin functions (Crest et al., 2017; Dai et al., 2018; Hollfelder et al., 2014; Urbano et al., 2009; Zang et al., 2015). Important effectors of integrins are Talin, which interacts with the actin cytoskeleton, and the pseudokinase integrin-linked kinase (ILK) which interacts with microtubules [MTs, (Akhtar and Streuli, 2013; Klapholz et al., 2015; Levi et al., 2006; Zervas et al., 2001)]. Interfering with integrin-mediated cell adhesion leads to morphological defects in many epithelia (Ambrosini et al., 2019; Fernandes et al., 2014; Goodwin et al., 2016; Ismat et al., 2013; Levi et al., 2006; Pastor-Pareja and Xu, 2011; Schöck and Perrimon, 2003) and conditions that affect bECM composition result in severe morphogenetic defects (Barqué et al., 2020; Dor-On et al., 2017; Jones et al., 2020; Messal et al., 2019; Thomas et al., 2018).

How cells establish apicobasal polarity depends largely on bECM interactions; therefore, these influence other aspects of cell physiology like vesicle transport and bulk membrane delivery (Denef et al., 2008; Devergne et al., 2014; Mathew et al., 2020; Vanderploeg et al., 2012; Yamazaki et al., 2016). bECM also influences cell shape changes, but how apical remodelling is mechanically coupled to bECM adhesion and how morphogenetic forces are transmitted between the two cellular domains are not fully resolved. Pioneering works have shown that apicobasal crosstalk and coordination are critical during morphogenesis, for example, for epithelial cell reorganisation during *Drosophila* germ-band elongation, and for endoderm invagination during ascidian development (Sherrard et al., 2010; Sun et al., 2017). But the subcellular players that mediate this coordination vary significantly across model systems. The actin cytoskeleton and MTs are generally the key players mediating these interactions, with varying contributions, organisation, and regulatory modules.

Here we review these issues by looking at two well-characterised morphogenetic processes that take place during *Drosophila* development: tracheal subcellular tube formation and apical oscillations of amnioserosa cells during dorsal closure. In the former, a wide landscape of mechanisms contributes to coordinate apical remodelling with bECM attachment, whereas in the latter, mechanisms of such crosstalk are less obvious. With these models in mind, we analyse how apicobasal crosstalk is established in other morphogenetic processes to identify the more general aspects of interaction between the two compartments.

## Apicobasal growth coordination during subcellular tube formation

**Fig. 1.**
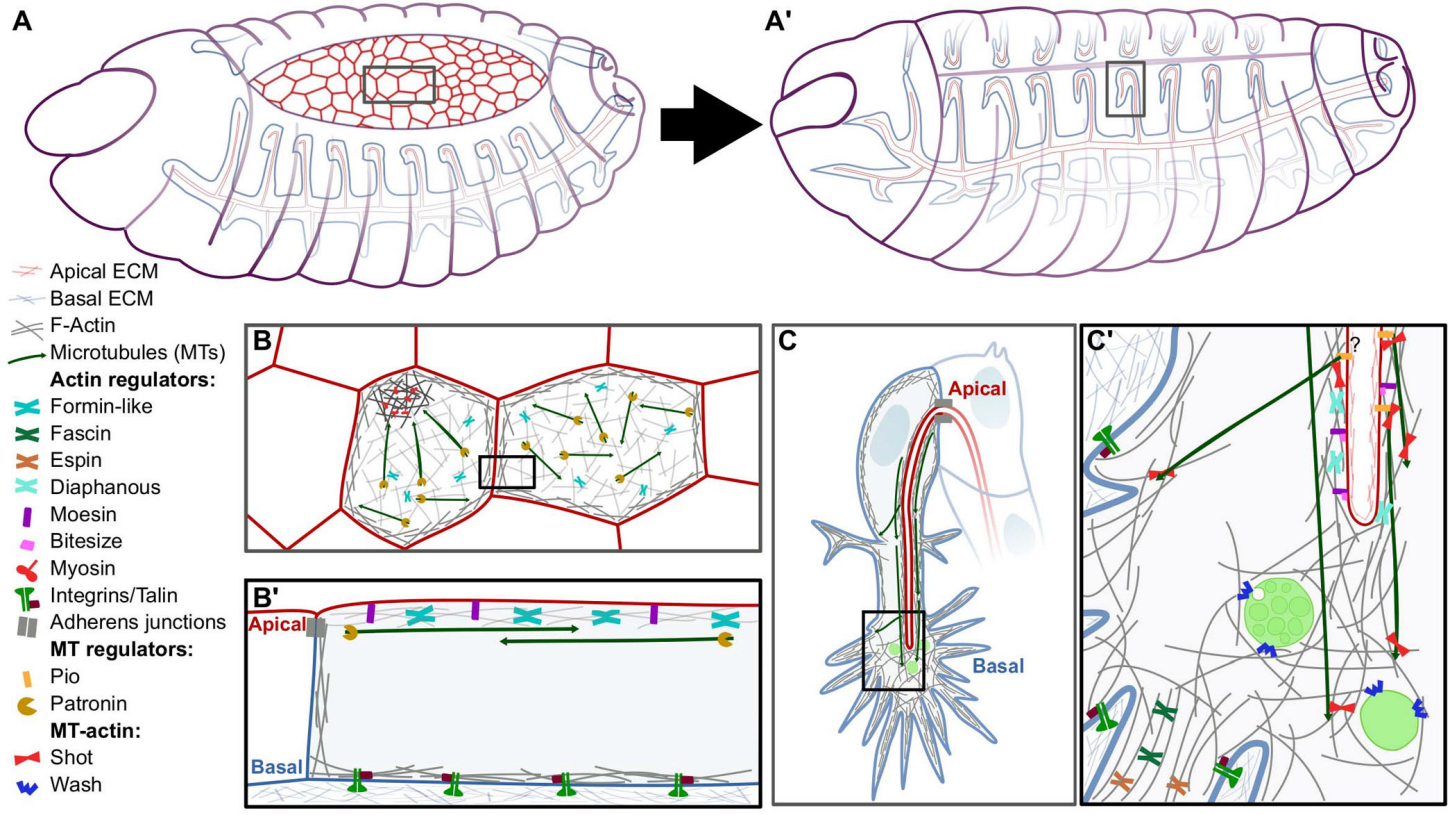
**Dorsal closure and subcellular tube formation as models of apicobasal interactions.** (A-A′) Illustrations of *Drosophila* embryos at developmental stages 14 (A) to 16 (A′), depicting dorsal closure and tracheal development. Apical and basolateral membranes are represented in red and blue, respectively. Squared box in (A) is magnified in (B) and squared box in (A′) is magnified in (C). (B) Zoom-in to two amnioserosa cells, viewed from their apical surface. The squared box is magnified in (B′) and shown as a cross-section. (C) Overview of a stage 16 tracheal terminal cell. The squared box is magnified in (C′). (C′) Zoom-in to the tip of a tracheal terminal cell. Question mark refers to the role of Pio in subcellular tube formation. Elements illustrated in the figure are listed to the left.

The complex morphology of the *Drosophila* tracheal system is very sensitive to perturbations, allowing straightforward identification of genes involved in its development. The finest tubes of the system lie at the tips of tracheal branches and are built by so-called terminal cells (Samakovlis et al., 1996). These subcellular tubes form by invagination of the apical plasma membrane of the terminal cells, allowing the tracheal lumen to grow inwards as the cell elongates (Fig. 1; Gervais and Casanova, 2010). The cell and its tube grow at very similar rates (JayaNandanan et al., 2014), making tracheal terminal cells particularly useful to study mechanisms of interaction between the apical and basal plasma membranes. Also, the mechanisms of terminal cell lumenogenesis have been shown to operate in the building of other tubular structures across the animal kingdom, such as the *Caenorhabditis elegans* excretory cell and vascular capillaries in vertebrates (Abrams and Nance, 2021; Bär et al., 1984; Cohen et al., 2020b; López-Novoa and Bernabeu, 2010).

*De novo* subcellular tube formation initiates in the embryo, and during larval development, the cell and its tube continue branching, forming ∼25 branches by the third larval instar (JayaNandanan et al., 2014). Each branch consists of a cellular protrusion containing a single ramification of the subcellular tube. Together, embryonic and larval studies provide us with a robust view of the cellular elements that coordinate apical and basal membrane extension. Failures in proper apicobasal interactions manifest as an uncoupling in the growth of the two membrane compartments, from absent subcellular tubes to tubes curling within cytoplasmic branches (JayaNandanan et al., 2014; Jones et al., 2014; Levi et al., 2006; Schottenfeld-Roames et al., 2014). These phenotypes allow us to understand how different mechanisms come together to allow proper subcellular tube formation.

Initial subcellular tube formation is driven by centrosomes near the apical compartment, which organise MTs to drive apical membrane invagination (Ricolo et al., 2016). Throughout subcellular tube growth, MTs run parallel to the tube with plus ends towards the growing tip of the cell (Gervais and Casanova, 2010; Schottenfeld-Roames and Ghabrial, 2012). Coordinated growth requires the interplay of actin and MTs; while actin is organised into different networks throughout the cell, MTs link these actin pools to coordinate their behaviour. Therefore, disrupting MTs by expression of the MT-severing protein Spastin uncouples directed growth of the apical compartment and results in guidance defects (Gervais and Casanova, 2010). Disrupting actin organisation in any subcellular compartment also prevents coordinated apicobasal growth (Gervais and Casanova, 2010; JayaNandanan et al., 2014; Levi et al., 2006; Okenve-Ramos and Llimargas, 2014; Ríos-Barrera and Leptin, 2021 preprint).

Organisation of the actin cytoskeleton requires the function of several proteins recruited at specific compartments. The apical actin cortex requires two pathways: one that depends on diaphanous (Dia), a crosslinker of the family of formins (Massarwa et al., 2009; Rousso et al., 2013), and one that uses the Ezrin-Radixin-Moesin (ERM) protein Moesin (Moe) along with its regulators Bitesize (Btsz), a synaptotagmin-like protein that recruits active Moe apically (JayaNandanan et al., 2014), and Slik, a kinase that mediates Moe activation (Ukken et al., 2014). In addition to the apical actin cortex, subcellular tube guidance depends on the basal plasma membrane. In this compartment, actin interacts with integrins and Talin, which presumably link the cell cytoskeleton to the bECM (Levi et al., 2006).

Another actin-based structure involved in subcellular tube formation lies in the space between the tip of the subcellular tube and the cell-growth cone, and it is referred to as the actin core (Gervais and Casanova, 2010; Okenve-Ramos and Llimargas, 2014; Oshima et al., 2006). We recently showed that late endosomes are responsible for the formation of this structure by promoting actin nucleation through Wash, a Wiscott-Aldrich Syndrome Protein (WASP) member (MacDonald et al., 2018; Mathew et al., 2020; Nagel et al., 2017; Ríos-Barrera and Leptin, 2021 preprint). Late endosomes and the associated actin core precede subcellular tube growth and branching, and inducing their mislocalisation affects directed apical membrane growth. Therefore, it is likely that endosome-associated actin ahead of the tube is required to coordinate subcellular tube and cell growth (Ríos-Barrera and Leptin, 2021 preprint). This model agrees with recent work from Ricolo and Araujo (2020) who have shown that Shortstop (Shot), the only member of the Spectraplakin family in *Drosophila*, is recruited to the different actin pools in the cell including the actin core, an essential function for proper tube formation. Shot crosslinks actin and MTs, therefore, it is responsible for bridging the distinct actin pools within the cell through MTs, stabilising subcellular tube growth and coupling it with cell elongation (Ricolo and Araujo, 2020; Fig. 1).

Together, the data discussed so far explain how actin pools and MTs collaborate to guide subcellular tube elongation with respect to the growing basal plasma membrane. Besides actin and MTs, the apical ECM (aECM) is also required to coordinate apicobasal behaviour. Affecting the synthesis of chitin, one of the major constituents of the aECM, leads to severe tube discontinuities and tortuosities in larval terminal cells (Rosa et al., 2018), but the intracellular mediators of these effects have not been identified. Other prominent components of the aECM are proteins bearing Zona Pellucida domains, also referred to as ZP proteins, and several of these have been studied in tracheal cells (Bökel et al., 2005; Caviglia et al., 2016; Jaźwińska et al., 2003; Sakaidani et al., 2011). Terminal cells express at least two of these: Dumpy (Dp) a secreted molecule, and Piopio (Pio), a transmembrane protein that can bind to MTs (Bökel et al., 2005; Rios-Barrera et al., 2017). However, even though these molecules are essential for the morphogenesis of other tissues (discussed below), knockdown experiments suggest that they are not required for subcellular tube formation in terminal cells (Rios-Barrera et al., 2017).

In conclusion, current evidence suggests that terminal cells possess redundant mechanisms to ensure a robust interaction between the apical and basal plasma membranes. This is reflected by the involvement of multiple cytoskeletal crosslinkers and ECM regulators that may provide at least partial redundancy to ensure proper apicobasal coordination (summarised in Fig. 1C-C′).

## The bECM and its impact on apical shape oscillations in the amnioserosa during dorsal closure

Dorsal closure is a morphological rearrangement taking place halfway during *Drosophila* embryogenesis. In this process, two opposing lateral epidermal sheets stretch and meet at the dorsal midline of the embryo, covering an eye-shaped epithelium called amnioserosa (Fig. 1). Originally considered passive players of dorsal closure, the cells of the amnioserosa are now known to generate the forces that promote stretching of the epidermis (Pasakarnis et al., 2016; Scuderi and Letsou, 2005; Wells et al., 2014). At the onset of dorsal closure, amnioserosa cells show a stochastic, pulsatile behaviour driven by apical actomyosin networks (David et al., 2010; Solon et al., 2009). Apical actomyosin accumulation leads to an acute reduction in the cell apical surface; upon resolution of the actomyosin foci, cells relax and expand apically (Blanchard et al., 2010; Duque and Gorfinkiel, 2016). Since these pulses are also asynchronous, during this phase the amnioserosa shows no net change in its area. In a second phase, actomyosin pulses remain stochastic but they gradually produce a net decrease in the apical surface of the whole tissue, a process that coincides with the stretching of the epidermis (Solon et al., 2009; Sumi et al., 2018). Therefore, as closure proceeds, interactions within cells of the amnioserosa through adherens junctions, and between the amnioserosa with the bECM and the epidermis, are critical for proper force propagation and coordination (Flores-Benitez and Knust, 2015; Goodwin et al., 2016; Jurado et al., 2016; Narasimha and Brown, 2004).

The amnioserosa lies on top of the yolk cell, and attachment between them via integrins and a bECM rich in laminin is essential for proper dorsal closure (Narasimha and Brown, 2004). Increasing or decreasing amnioserosa cell adhesion to the bECM alters the rate of dorsal closure because both conditions perturb the optimal propagation of forces within the amnioserosa. Artificially increasing tension by expressing an overactive form of Talin results in decreased shape oscillations, low force transmission to neighbouring cells, and in consequence, inefficient epithelial remodelling. Conversely, cells that lack β-Integrin show very pronounced apical surface oscillations but the forces these cells generate are propagated to more cells than in control embryos, albeit inefficiently (Goodwin et al., 2016). Cells mutant for β-Integrin also show aberrant E-Cadherin distribution and turnover, together with altered apical actomyosin dynamics that explain the defects in apical pulsations (Goodwin et al., 2017; Jurado et al., 2016; Meghana et al., 2011; Saravanan et al., 2013). Given that E-Cadherin also interacts with the actin cytoskeleton and that it is required for proper force transmission across cells, both components, cell–cell and cell–ECM adhesion should be coordinated to allow amnioserosa cell pulsation.

In contrast to subcellular tube formation, what mediates the crosstalk between the basal plasma membrane and the apical compartment in amnioserosa cells is not immediately obvious. While in tracheal terminal cells many mediators of this crosstalk have been described, these do not seem to be required for amnioserosa shape changes. This might be explained by the topological differences between amnioserosa and tracheal terminal cells. Amnioserosa cells are large, almost flat cells, with outspread apical and basal surfaces. In contrast, tracheal terminal cells completely invaginate their apical compartment to build a subcellular tube. Amnioserosa cells concentrate most of their MTs at the apical cortex, parallel to the plasma membrane. There, they favour the organisation of actomyosin pulses, suggesting they support apical membrane organisation rather than apicobasal coordination (Fig. 1B; Guru et al., 2021 preprint; Meghana et al., 2011; Pope and Harris, 2008). Further support of this comes from perturbation experiments; if MTs mediate the apicobasal coordination in amnioserosa cells, altering MT dynamics should result in similar phenotypes as the ones caused by loss of integrins. However, preventing MT growth by expression of a dominant negative form of EB1 or by overexpression of Spastin leads to the opposite effect, with decreased apical shape fluctuations and actomyosin pulses (Guru et al., 2021 preprint). Shot, one of the main regulators of apicobasal crosstalk in tracheal terminal cells, participates in dorsal closure by regulating filopodia formation in the epidermal leading edge cells, consistent with the polarised distribution of MTs in these cells (Gomez et al., 2016; Takács et al., 2017), but currently no role for Shot in the amnioserosa has been reported.

Besides forming actomyosin pulsing networks, the apical domain of amnioserosa cells is shielded by a persistent actin cortex that is required for proper apical oscillations (Dehapiot et al., 2020). The actin crosslinker Formin-like (Fmnl) organises this cortex, which coexists with actomyosin pulses throughout dorsal closure. Knocking down Fmnl results in wider actomyosin pulses and greater apical area oscillations, a response that is also seen upon loss of bECM attachment. In addition, similar to increasing bECM adhesion, overexpression of Fmnl leads to reduced apical pulsations (Dehapiot et al., 2020; Goodwin et al., 2016). These results argue in favour of cortical actin directly mediating the interaction between the apical and basal plasma membranes. Other studies have shown that changes in tension during apical oscillations alter actin dynamics, for instance, at cell junctions to ensure their integrity (Hara et al., 2016; Sumi et al., 2018). Apicobasal crosstalk could therefore be regulated by stable actin networks at the apical and basal plasma membranes that are then crosslinked by junctional actin, which is constantly buffered by mechanosensation. In agreement with this, altering Moesin apical recruitment greatly affects actomyosin organisation in the apical compartment and at the adherens junctions, leading to higher amplitude actomyosin pulses (Flores-Benitez and Knust, 2015). Altogether, these results suggest that cortical and junctional actin pools communicate with each other, allowing coordination of apicobasal behaviour.

## Other mechanisms of apicobasal crosstalk

Subcellular tube formation has shown us that multiple pathways can collaborate to ensure proper apicobasal coordination, but as illustrated by amnioserosa apical oscillations, these mechanisms can vary significantly in other contexts. In the following, we will summarise the crosstalk principles that have been described in other morphogenetic rearrangements and the molecules that mediate them.

### Crosslinking actin cortices through MTs

**Fig. 2.**
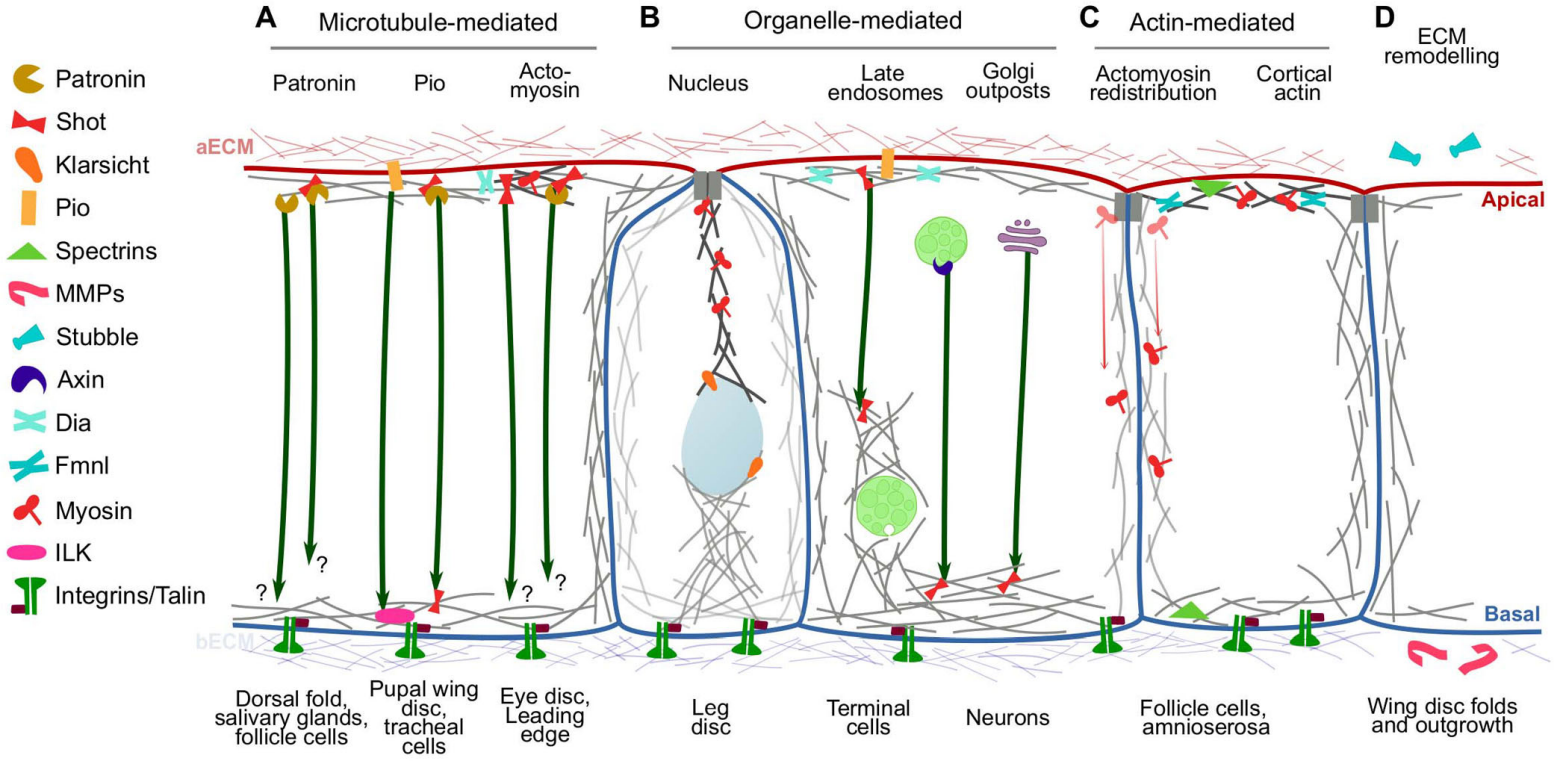
**Different mechanisms of apicobasal crosstalk in *Drosophila.*** Interactions between bECM and the apical compartment in different systems. Apical and basolateral membranes are represented in red and blue, respectively. Processes where each mechanism has been observed are indicated at the bottom. MTs are represented as arrows; MT plus ends being the arrowheads. Curved grey lines symbolize F-actin; those accompanied with Myosin are actomyosin. (A) MT-based crosstalk. Patronin participates in the three described processes, whereas Shot is not required for dorsal fold formation. Question marks indicate processes where basal interactors have not been identified. (B) Organelle-based crosstalk. (C) Actin-based crosstalk. Gradient red arrows indicate Myosin movement. (D) ECM remodelling. Elements illustrated in the figure are listed to the left.

In many morphogenetic events in Drosophila and other organisms, non-centrosomal MTs connect local cell-shape changes to distant positions through specific adaptors (Lee and Harland, 2007; Lee et al., 2007; Yano et al., 2021). Many tissues orient MTs with minus ends toward the apical compartment and the plus ends toward the basal, and this organization is held through MT-binding adaptors (Fig. 2A). One of the very versatile MT regulators is Shot, which depending on the context, gets recruited basally, apically, or in both compartments, and depending on its binding partners, it can interact with MT plus or minus ends. Shot recruitment at MT minus ends is mediated by Patronin, and its plus end localization is mediated by EB1 (Applewhite et al., 2010; Booth et al., 2014; Ghislain et al., 2021; Goodwin and Vale, 2010; Lee et al., 2016; Mimori-Kiyosue et al., 2000; Molines et al., 2018; Nashchekin et al., 2016). Despite the different scenarios in which Shot can regulate MTs and their interaction with apicobasal compartments, its contribution to a given process can vary drastically. For instance, in late pupal wing development, MTs run along the apicobasal axis and they stabilise adhesion to the apical and basal ECMs. Shot is found at both ends, as it is in terminal cells. However, loss of Shot has no effect on MT architecture or epithelial morphology. Instead, in this tissue, MTs are stabilised through their interactions with Pio in the apical membrane and ILK and integrins in the basal domain (Akhtar and Streuli, 2013; Bökel et al., 2005). These results contrast with subcellular tube formation, where Shot seems to contribute more than Pio in mediating apicobasal interactions (Jaźwińska et al., 2003; Ricolo and Araujo, 2020). Shot and Patronin organise MTs at the apical compartment in a range of processes, like in the formation of apical actin-based microvilli of follicle cells and in salivary glands, where they are required for proper tissue invagination (Booth et al., 2014; Ghislain et al., 2021; Khanal et al., 2016; Röper, 2012). In these models, MT apical anchoring is required to sustain novel architectures, but whether they interact with the basal plasma membrane is still unknown.

There are processes that use MT apical anchoring to drive cell-shape changes but do not seem to depend on Shot at all. This is seen for instance in dorsal fold formation during early development, where instead, Patronin organises an MT apical cortex that is required to initiate fold formation. In this process, instead of a transversal array, MTs form an apical dome that allows proper tissue folding (Takeda et al., 2018). Similarly, a morphogenetic furrow regulates proper photoreceptor organisation in the eye disc (Ready et al., 1976). This furrow is formed through apical recruitment of actomyosin which induces apical constriction, and apicobasal MTs are also required for the shape change. Loss of integrins affects MT organisation and furrow formation, but the elements that mediate MT anchoring to the apical and basal compartments have not been reported, and at least involvement of ILK has been ruled out (Corrigall et al., 2007; Fernandes et al., 2014).

### Organelle-mediated crosstalk mechanisms

To coordinate apical and basal behaviour, some tissues have adapted mechanisms that involve intracellular relay points (Fig. 2B). Leg disc development uses apoptosis as pulling force to induce fold formation (Manjón et al., 2007). In this process, cells prompted to die first reduce their apicobasal axis, a process that requires nuclear repositioning toward the basal compartment and actin reorganization around the nucleus. The nucleus mediates the interactions between the apical and basal compartments; it interacts apically with an actomyosin network, and it is associated to F-actin and Talin/integrins on the basal compartment. Disturbing actomyosin by using laser cuts or affecting basal anchoring by removing Talin prevent proper force transmission from the apical to the basal compartment. Evidence for the involvement of the nucleus comes from experiments on Klarsicht, a KASH domain protein that connects the cytoskeleton to the nuclear envelope. Loss of Klarsicht prevents cell shortening, nuclear repositioning and fold formation, showing that both the nucleus and the actin around it are required for proper cell deformation (Ambrosini et al., 2019).

Klarsicht also interacts with MTs, a role required for nuclear positioning in other cells like differentiated photoreceptors (Fischer et al., 2004; Mosley-Bishop et al., 1999). In salivary glands, Klarsicht favours collective cell migration by regulating MT organisation (Myat and Andrew, 2002; Myat et al., 2015). Klarsicht expression is enriched at the salivary gland placodes (Myat and Andrew, 2002). It is not known whether MTs are anchored basally during placode invagination, but it is an intriguing possibility that Klarsicht and the nucleus could have a role in this process, as they do during leg development.

Late endosomes have various roles in migration and cytoskeletal organisation throughout metazoans (MacDonald et al., 2018; Palamidessi et al., 2008; Ramírez-Santiago et al., 2016; Schiefermeier et al., 2014). As mentioned for subcellular tube formation in tracheal terminal cells, late endosomes can also mediate interactions between distant plasma membrane domains by regulating the cytoskeleton (Ríos-Barrera and Leptin, 2021 preprint). Regulation of the cytoskeleton by endosomes has been documented in other processes: peripheral sensory neurons use endosomes as MT organising centres (MTOCs) at branching points, which allows MT growth towards dendritic termini. This function is mediated by Axin, a component of the Wnt signalling pathway that can interact with γ-Tubulin. Loss of Axin results in reduced branching, and targeting Axin to mitochondria is sufficient to induce MT reorganization (Weiner et al., 2020). Golgi outposts have also been proposed as MTOCs that mediate neuronal branching in Drosophila and mammalian systems (Du et al., 2021a; Fu et al., 2019; Ori-McKenney et al., 2012; Ye et al., 2007). However, presence of Golgi and MT minus-end markers does not seem to correlate in various experimental setups, which has brought MT nucleation at Golgi outposts into question (Nguyen et al., 2014; Weiner et al., 2020). Further ultrastructural analyses or other studies should define whether Golgi outposts can indeed mediate MT nucleation. Also, more works are required to determine how widespread organelle-mediated apicobasal crosstalk mechanisms are.

### Direct apicobasal force propagation through cortical actin

The actin cytoskeleton forms cortical arrays throughout the surface of the cell (Fig. 2C). Remodelling of these pools can also propagate forces to distant subcellular compartments promoting cell shape changes, as experiments in the amnioserosa suggest. This has been also observed in larval wing disc development; first, in a transition from cuboidal to columnar, wing disc cells concentrate actomyosin in the apicolateral compartment, which decreases cortical tension basally. This allows cells to elongate in the apicobasal axis. In this process, severing MTs has no effect on the cuboidal to columnar transition, further reinforcing the relevance of cortical actin in the shape change (Widmann and Dahmann, 2009). Later, the wing epithelium folds to form a central pouch. Fold formation is again regulated by actomyosin redistribution. One of the folds is formed by basal relaxation of the actin cytoskeleton accompanied by bECM degradation, whereas the other fold is formed by increased lateral tension generated by actomyosin redistribution (Sui et al., 2018). Similar cortical actin rearrangements control the elongation of the pseudostratified epithelium of the zebrafish retina, which, together with proliferation, allows the whole tissue to grow (Matejčić et al., 2018).

Cortical actin also participates in the elongation of the follicular epithelium, and in this case spectrins at both compartments are responsible for organising actin. Loss of basal spectrins or integrins prevents proper cuboidal to columnar transition, with general actin disorganization (He et al., 2010; Santa-Cruz Mateos et al., 2020; Ng et al., 2016; Qin et al., 2017). Loss of integrins also affects the formation of actomyosin networks in the apical and basal compartments (He et al., 2010; Santa-Cruz Mateos et al., 2020; Qin et al., 2017). Thus, follicle cells resemble the amnioserosa, in that both have persistent actin cortices (mediated by Fmnl in the amnioserosa and spectrins in follicle cells) and actomyosin pulses that are dependent on proper integrin function. As mentioned earlier, follicle cells also use MTs to stabilise their apical architecture, although it is not known if these MTs are stabilised basally. Why some tissues require MT crosslinking the apical and basal compartments while others rely solely on cortical actin is not immediately clear, but the answer might be in the geometry of cells or in tissue-scale forces and how they influence cytoskeletal organization.

### Cell-shape changes driven by aECM remodelling

aECMs cover many epithelia, particularly within tubular structures like the vertebrate vasculature, lungs and kidneys, the *C. elegans* vulva and excretory cell, and the *Drosophila* tracheal system. While we have discussed the role of Pio as a link between MTs and the aECM, recent works in *Drosophila* and *C. elegans* have revealed multiple ways in which this matrix influences morphogenesis. ZP proteins like Pio are very abundant constituents of the aECM, with 43 ZP genes in *C. elegans* and 20 in *Drosophila*. These molecules are able to form complex web-like multimers through their ZP domains, which similar to the bECM, provide a scaffold that drives and stabilises cell shape changes (Cohen et al., 2019, 2020a,b; Itakura et al., 2018; Ray et al., 2015; Smith et al., 2020).

Experiments in tracheal multicellular tubes have shown multiple ways in which the aECM contributes to morphogenesis. Conditions that increase aECM deposition or that reduce it result in reduction or expansion of apical surfaces, respectively (Dong et al., 2014a,b; Öztürk-Çolak et al., 2018). Additionally, whereas apical actin bundles instruct supracellular organisation and synthesis of the aECM, the aECM also feeds back into the cells to reinforce actin bundle and adherens junctions organisation (Öztürk-Çolak et al., 2016b). Direct effects on the organisation of the basal compartment have not been reported, nevertheless, changes in apical architecture affect the overall shape of the tracheal multicellular tubes. The role of ZP proteins in shaping tubes is conserved in vertebrates; Endoglin, a transmembrane ZP protein, is involved in angiogenesis in zebrafish and in mice (Sugden et al., 2017). Furthermore, in human patients, mutations in Endoglin results in hereditary haemorrhagic telangiectasia, a condition that affects vascular morphology and leads to haemorrhages (McAllister et al., 1994). Endoglin also interacts with MT-associated proteins, suggesting that Pio's function in MT organisation is also conserved (Meng et al., 2006).

Further evidence of the role of the aECM in apicobasal crosstalk comes from experiments in the pupal wing disc. As mentioned earlier, during larval development the wing disc epithelium transitions from cuboidal to cylindrical in a process that requires reorganisation of the cortical actin cytoskeleton. In pupal development, wing disc eversion requires a transition back from columnar to cuboidal together with convergent extension to allow the elongation of the wing. This is achieved by coordinated secretion of Stubble (Sb), a protease that degrades the aECM, and of Matrix Metalloprotease 2 (MMP2), which degrades the bECM. As in tracheal multicellular tubes, aECM remodelling also reorganises the actin cytoskeleton, which couples the matrix reorganization with cell shape changes (Diaz-de-la-Loza et al., 2018; Ray et al., 2015).

## Conservation of apicobasal coordinators: examples beyond the animal kingdom

We have described various mechanisms that allow coordinated behaviour between the apical and basal compartments during cell-shape changes. While we focused our analyses on *Drosophila* development, these mechanisms also operate in the morphogenesis of other animals and most proteins discussed are conserved across the animal kingdom (Table 1). However, cell shape changes participate in the development of most organisms, therefore, some of these principles could have more ancestral functions. For instance, in filamentous fungi, growth of hyphal tips is regulated by polarised organisation of actin and MTs that generate force and transport molecules to the growing tip. This is coordinated by a collection of secretory vesicles known as Spitzenkörper, which provide membrane material to the growing tip and also allow anchoring of MTs and actin, as late endosomes do during tracheal terminal cell development (Crampin et al., 2005; Ríos-Barrera and Leptin, 2021 preprint; Zheng et al., 2020). In addition, similar to late endosomes in subcellular tube formation, Spitzenkörper relocalisation precedes changes in the direction of hyphal growth (Crampin et al., 2005).

**Table 1.**
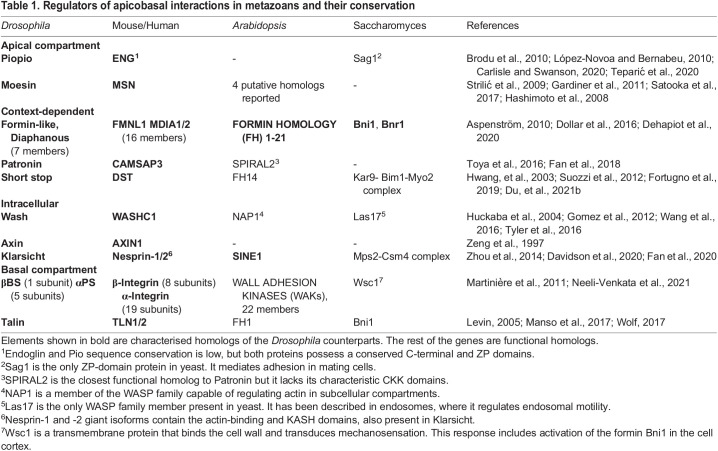
Regulators of apicobasal interactions in metazoans and their conservation

Adhesion complexes in plants also reveal intricate modes of functional conservation across eukaryotes. Even though plants do not possess integrins, they also interact with the cell wall matrix and transduce mechanical information intracellularly. Among other mechanosensors, plants use Formin Homology (FH) proteins to interact with the cell wall. Arabidopsis possesses 21 FH genes, and they all carry out distinct functions; FH1 is a transmembrane protein that binds the ECM extracellularly and regulates cortical actin organisation intracellularly (Wolf, 2017). Yeasts also use formins for various processes; interaction with the cell wall is mediated by the transmembrane protein Wsc1, which, in turn, activates the formin Bni1 to regulate the actin cytoskeleton (Levin, 2005). In filamentous fungi, the Spitzenkörper also recruits Bni1/Formin homologs, which organise the actin cytoskeleton around these vesicles (Zheng et al., 2020). As discussed above, formins in animals have typically been recognised for their roles as actin regulators, as is the case for Dia in subcellular tube formation and for Fmnl in amnioserosa apical shape oscillations. However, *Drosophila* Formin3 and Dishevelled-associated activator of morphogenesis (DAAM) interact with MTs and actin during neuronal branching (Das et al., 2021; Szikora et al., 2017). The role of formins as actin-MT regulators also goes beyond animals; *Arabidopis* FH14 is another formin capable of interacting with MTs (Du et al., 2021b). Together, these works suggest that the involvement of formins in execution and stabilisation of shape changes is highly conserved.

## Concluding remarks and open questions

Here we summarised a range of mechanisms that cells and tissues use to coordinate remodelling of their apical and basal compartments. We show that actin and MT regulators (1) can be recruited differently depending on the context, (2) can act in parallel to other mechanisms increasing redundancy, and (3) that their relative contribution to a process can vary greatly from one context to the other. For some of the systems described here there is plenty of information on how multiple pathways convey to regulate apicobasal interactions, like the case of subcellular tube formation, wing and leg disc development, and follicle cell elongation. In other models, some gaps still need to be filled in. We focused our work on the role of integrins and Talin, but other basal complexes could also be involved in mediating apicobasal crosstalk. This is the case of the Dystroglycan/Dystrophin complex, which contributes to cytoskeletal reorganisation in different scenarios (Alégot et al., 2018; Campos et al., 2020). It will also be interesting to see how variations in ECM composition and stiffness can affect apical membrane remodelling, how these changes are translated intracellularly, and how they intersect with the bECM as signalling scaffold (Chen et al., 2019; Crest et al., 2017; Ma et al., 2017).

While this work focuses on the impact of the bECM in apical morphogenesis, recent evidence suggests that the apical membrane domain can also signal back to the basal compartment and influence bECM properties. In follicle cells, the apical determinant Phosphatidylinositol 4,5-bisphosphate (PIP_2_) is required to restrict Rab10 activity to the basal compartment of the cell. Rab10 is a GTPase involved in secretion of bECM molecules like Collagen IV and Perlecan, and in conditions that reduce PIP_2_ abundance, Rab10 can also secrete bECM molecules towards the apical compartment (Devergne et al., 2014; Lerner et al., 2013; Román-Fernández et al., 2018). In contrast to the follicular epithelium, many tissues rely on others to assemble their apical and basal ECMs (Dong et al., 2014b; Martinek et al., 2008; Matsubayashi et al., 2017; Pastor-Pareja and Xu, 2011; Rios-Barrera et al., 2017), but it is likely that fine-tuning of ECM properties is regulated cell-autonomously by secretion of proteases as has been shown in the pupal wing disc (Diaz-de-la-Loza et al., 2018). Finally, we point readers to other reviews that elaborate on specific aspects related to this work (Table 2).

**Table 2.**
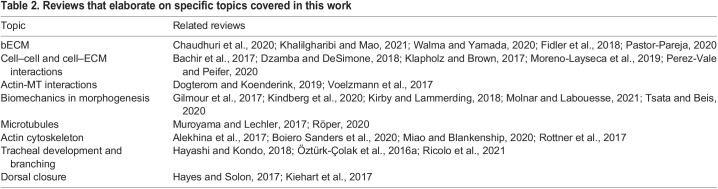
Reviews that elaborate on specific topics covered in this work
